# Benefits and harms of 4-factor prothrombin complex concentrate for reversal of vitamin K antagonist associated bleeding: a systematic review and meta-analysis

**DOI:** 10.1007/s11239-017-1506-0

**Published:** 2017-05-24

**Authors:** Marjolein P. A. Brekelmans, Kim van Ginkel, Joost G. Daams, Barbara A. Hutten, Saskia Middeldorp, Michiel Coppens

**Affiliations:** 1 Department of Vascular Medicine, Academic Medical Center, Meibergdreef 9, 1105 AZ Amsterdam, the Netherlands; 2 University of Amsterdam, Amsterdam, the Netherlands; 3 Medical Library, Academic Medical Center, Amsterdam, the Netherlands; 4 Department of Clinical Epidemiology, Academic Medical Center, Amsterdam, the Netherlands

**Keywords:** Vitamin K antagonist, Bleeding, Prothrombin complex concentrate, Fresh frozen plasma, INR normalization, Mortality

## Abstract

Prothrombin complex concentrate (PCC) is used for reversal of vitamin K antagonists (VKA) in patients with bleeding complications. This study aims to assess benefits and harms of 4-factor PCC compared to fresh frozen plasma (FFP) or no treatment in VKA associated bleeding. PubMed, EMBASE and CENTRAL were searched from 1945 to August 2015. Studies reporting 4-factor PCC use for VKA associated bleeding and providing data on INR normalization, mortality or thromboembolic (TE) complications were eligible. Two authors screened titles and full articles for inclusion, extracted data, and assessed risk of bias. Mortality data were pooled using Mantel–Haenszel random effects meta-analysis. Nineteen studies were included (N = 2878); 18 cohort studies and one RCT. Six studies had good methodological quality, 9 moderate and 4 poor. Baseline INR values ranged from 2.2 to >20. The INR within 1 h after PCC administration ranged from 1.4 to 1.9, and after FFP administration from 2.2 to 12. PCC reduced the time to reach INR correction in comparison with FFP or no treatment. The observed mortality rate ranged from 0 to 43% (mean 17%) in the PCC, 4.8–54% (mean 16%) in the FFP and 23–69% (mean 51%) in the no treatment group. Meta-analysis of mortality data resulted in an OR of 0.64 (95% confidence interval [CI] 0.27–1.5) for PCC versus FFP and an OR 0.41 (95% CI 0.13–1.3) for PCC versus no treatment. TE complications were observed in 0–18% (mean 2.5%) of PCC and in 6.4% of FFP recipients. Four-factor PCC is an effective and safe option in reversal of VKA bleeding events.

**Electronic supplementary material:**

The online version of this article (doi:10.1007/s11239-017-1506-0) contains supplementary material, which is available to authorized users.

## Introduction

Vitamin K antagonists (VKA) are widely prescribed anticoagulant agents. Numerous trials have shown that these agents are effective in the prevention and treatment of acute and chronic venous and arterial thromboembolic diseases [1]. VKA therapy is challenging because of a narrow therapeutic window and the need for regular laboratory monitoring and dose adjustments [2, 3]. In addition, bleeding complications are a frequently observed side effect [3, 4]. Major bleeding episodes typically involve the gastrointestinal tract, urinary tract or intracranial sites and occur in 1–3% of VKA treated patients per year [5, 6]. Lethal hemorrhagic complications have an incidence of around 1% per year [5, 7]. The risk of major bleeding is associated with elevated international normalized ratios (INR); at an INR of 2.0 the bleeding risk is already increased and rises exponentially when the INR exceeds 5.0 [5, 7]. Therefore, it is no surprise that VKA top the list of medications that lead to hospital admissions [8].

Reversal of the anticoagulant effect of VKA may be required in patients with severe bleeding or in those who need to undergo an emergency invasive procedure. Depending on the clinical situation this reversal need to be completed within several hours or immediate [9]. The most straightforward intervention to counteract the effect of VKA is the administration of vitamin K [1, 10, 11]. When vitamin K is given intravenously, the INR will start to drop within 4 h of administration and will be normalized after 12–16 h [12]. Normalization of INR after oral administration of vitamin K will take up to 24 h [13].

Immediate reversal may be achieved by replacing deficient clotting factors [1, 2, 14]. In North America, fresh frozen plasma (FFP) used to be the most commonly used coagulation factor replacement product for reversal of VKA and contains factors II, VII, IX and X in a low concentration [15, 16]. Limitations of FFP use are risk of volume overload and transfusion reactions. Also, FFP is rarely able to normalize the INR completely [15–18]. Three-factor prothrombin complex concentrate (PCC) contains the vitamin K dependent coagulation factors (factors II, IX and X), as well as anticoagulant proteins C and S (and sometimes a small concentration of heparin). In addition, in 4-factor PCC preparations also coagulation factor VII is present [15, 19]. PCC provides a rapid and effective option for normalization of INR [20]. Other advantages are a reduced infusion volume and low risk of pathogen transmission [21]. In Europe, 4F-PCC has been available for decades for urgent reversal of VKA therapy. However, 4F-PCC was not approved until 2008 in Canada and 2013 in the USA, mainly due to the uncertainty of prothrombotic complications. Several international guidelines now recommend the use of PCC in reversal of VKA associated bleeding [1, 11, 22].

Several prospective studies and a few systematic reviews have been published regarding the effectiveness and safety of PCC in VKA reversal, mainly including bleeding but also non-bleeding patients requiring emergent invasive procedures, combining data on 3-factor and 4-factor PCC and predominantly addressing INR normalization or thromboembolic complications [6, 23–26]. The aim of this focused systematic review and meta-analysis was to evaluate the benefits and harms of 4-factor PCC, including INR correction, mortality, thromboembolic complications, functional outcomes and duration of hospitalization in patients with VKA associated bleeding complications. In addition, we assessed 4-factor PCC in comparison to FFP and no treatment.

## Materials and methods

This systematic review and meta-analysis was performed according to the PRISMA Statement [27]. A short study protocol was composed (in Dutch) but not published online.

### Search strategy

To identify all available articles on the use of 4-factor PCC in VKA related bleeding, we conducted a literature search in PubMed (1945–August 2015), EMBASE (1947–August 2015) and Cochrane Central Register of Controlled Trials (CENTRAL; 1976–August 2015) electronic databases. For PubMed and EMBASE a language filter was applied and a database specific strategy was adopted to exclude as many as possible animal studies. Details of the full search strategy can be found in Appendix I in the Supplement. Additionally, we manually searched references from four recent review articles [6, 23, 26, 28] which evaluated PCC for emergency reversal of VKA, but this revealed no new articles.

### Study selection

The selection process was divided into three successive stages: title-, abstract- and full manuscript selection. Two authors (MB, KG) independently assessed the eligibility of retrieved studies according to predetermined criteria. Difference in judgment was solved by discussion.

Inclusion was based on the following criteria: the use of 4-factor PCC in patients presenting with VKA associated bleeding; and reporting on any of the following: INR normalization, number of thromboembolic events within 30 days, mortality, functional outcomes or length of stay in ICU. We included all studies using 4-factor PCC, regardless of whether or not a comparator was present. Exclusion criteria were: animal experiments; in vitro and ex vivo studies; studies that included less than 5 patients; the use of 3-factor PCC or activated 4-factor PCC; studies involving the concomitant use of recombinant factor VIIa; and studies that were duplicate reports or preliminary reports of data later presented in full (including congress abstracts).

### Data extraction

Data of selected studies were extracted independently by two reviewers (MB, KG) on a structured data extraction form. Information was collected on: study design, number of patients, mean age of patients, type of bleeding, indication for PCC, type of PCC given, applied intervention, PCC dose, baseline INR, INR after intervention, ICU admission days, functional outcomes, follow-up days, mortality and thromboembolic (TE) complications.

### Study quality assessment

The methodological quality of the included studies was assessed independently by two authors (MB, KG) using the Cochrane Collaboration’s tool for assessing risk of bias [29] and the Newcastle-Ottawa scale assessment of cohort and case control studies [30]. Studies were checked for selection bias, information bias, and the general validity and quality of the studies. Disagreements were solved by discussion. We qualified a study as poor, moderate or good, depending on their scores on the appropriate checklist. Since the cut-off score for each category was not defined in the literature and was up to the discretion of the author, we decided to use the following classifications in consultation with a clinical librarian (JD) and a clinical epidemiologist (BH). The maximum score on the Newcastle-Ottawa scale is 9 points. Poor outcome was assigned when the study had less than 6 points. Moderate outcome was assigned to studies with 6 points whereas more than 6 points qualified for good. The maximum score on the Cochrane Collaboration’s tool for assessing risk of bias is 7 points. Poor outcome was assigned when the study had less than 4 points, moderate was assigned to studies with 4 points and more than 4 points qualified as good.

### Statistical analysis

Data are reported as mean (standard deviation [SD]), median (range), counts or percentages. Comparisons between the different treatment regimens (PCC, FFP and no treatment) were made using the Chi-square test for categorical variables (mortality, TE complications). A p-value of less than 0.05 was considered to be statistically significant. We performed an exploratory meta-analysis of the mortality data using Review Manager 5.3. The forest plots were visually examined and we assessed the statistical heterogeneity across the studies using the Cochran’s Q test and I^2^ values. We considered values of 25–50%, 50–75% and ≥75% to indicate low, moderate and high heterogeneity, respectively. In case of low heterogeneity, the odds ratios (OR) were combined across studies using the Mantel–Haenszel procedure which assumes a fixed treatment effect. When heterogeneity was moderate, study data were combined using a random effects model according to the method of Mantel–Haenszel.

## Results

### Search results

**Fig. 1:**
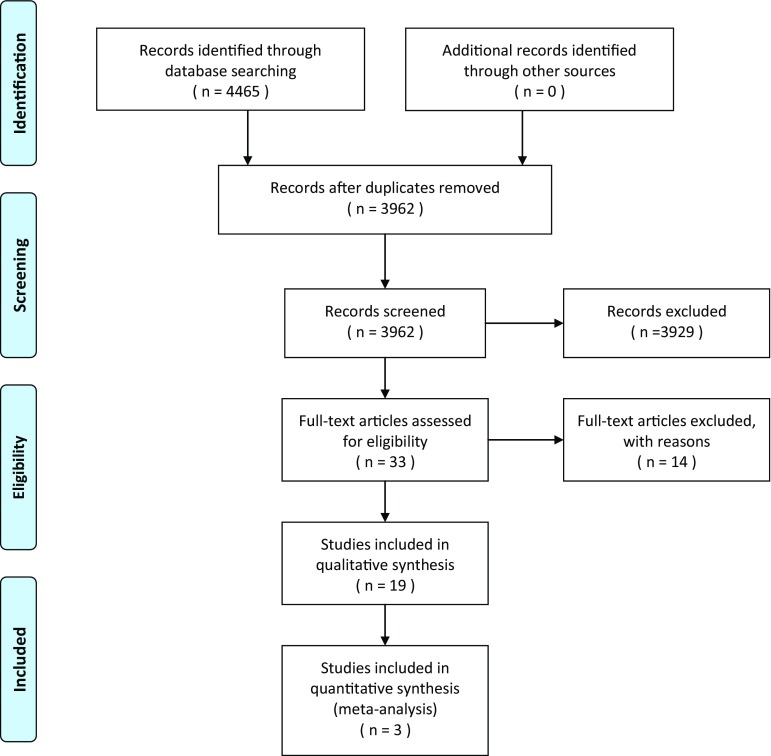
PRISMA flow diagram of the study selection process

The systematic search identified 3962 articles (Medline (n = 2530); Embase (n = 1358); and CENTRAL (n = 74); see Fig. 1). Title and abstract screening identified 49 studies that were evaluated in more detail. After full article screening, we included 19 studies with a total of 2878 participants [31–49].

### Characteristics of included studies

Characteristics of the included studies are shown in Table 1. Of these, 13 were prospective cohort and 5 retrospective cohort studies and one was a randomized controlled trial (RCT). The studies ranged in size from 10 to 822 patients and mean age ranged from 67 to 78 years. The most commonly observed bleeding complication was intracranial hemorrhage (ICH; 15 studies).

**Table 1:** Baseline characteristics of the studies

References	Study design	Study quality	Type of bleeding	Patients (n)	Mean age (years)	Type of PCC	Intervention
[34]	Prospective cohort	Poor	Extracranial	10	73	Beriplex	PCC (n = 10), weight based No control group
[48]	Prospective cohort	Moderate	ICH	17	69	PPSB-HT Nichiyaku	PCC (n = 17), fixed 500 or 1000 IU No control group
[40]	Prospective cohort	Poor	GI, hematuria	10	73	Octaplex	PCC (n = 10), INR + weight based No control group
[36]	Prospective cohort	Good	ICH	111	77	Proplex T	PCC (n = 46), INR based No treatment (n = 65)
[43]	Prospective cohort	Moderate	GI, subcutaneous, other	17	70 (median)	Beriplex	PCC (n = 17), INR + weight based No control group
[38]	Prospective cohort	Good	ICH	50	69	PPSB-HT Nichiyaku	PCC (n = 22), INR based No treatment (n = 28)
[33]	Prospective cohort	Moderate	ICH	141	78	Octaplex	PCC (n = 141), fixed 1000 IU No control group
[31]	Prospective cohort	Moderate	ICH Extracranial	174	76	Octaplex Kaskadil	PCC (n = 174), INR + weight based No control group
[35]	Retrospective cohort	Good	ICH	181	69	Cofact	PCC (n = 41), INR + weight based No treatment (n = 140)
[31]	Prospective cohort	Moderate	ICH, GI, other	646	78	Octaplex	PCC (n = 646), INR + weight based No control group
[41]	Prospective cohort	Moderate	ICH, GI, other	116	75	Octaplex Beriplex Prothromplex	PCC (n = 116), INR + weight based No control group
[39]	Retrospective cohort	Poor	ICH	22	68	Prothromplex	PCC (n = 22), fixed dose 15 IU/kg or INR based No control group
[44]	RCT	Good	GI, nonvisible, ICH and musculoskeletal	202	70	Beriplex	PCC (n = 98), INR + weight based FFP (n = 104)
[46]	Retrospective cohort	Moderate	ICH and extracranial	76	78	Octaplex	PCC (n = 76), fixed 1000 IU No control group
[42]	Retrospective cohort	Good	ICH	135	73	Cofact Octaplex Beriplex Prothromplex	PCC (n = 100), INR + weight based FFP (n = 35)
[47]	Prospective cohort	Moderate	ICH	33	–	Beriplex	PCC (n = 5), INR + weight based FFP (n = 28)
[45]	Prospective cohort	Moderate	ICH, GI, deep muscle and other	822	77	Kanokad Octaplex	PCC (n = 509), INR + weight based No treatment (n = 313)
[37]	Prospective cohort	Good	GI	40	67	Cofact	PCC (n = 20), INR + weight based FFP (n = 20)
[49]	Retrospective cohort	Poor	ICH, GI, muscle, urinary, respiratory, other	75	68	Cofact Kaskadil	PCC (n = 74), INR + weight based No control group

*ICH* intracranial hemorrhage, *PCC* prothrombin complex concentrate, *INR* international normalized ratio, *FFP* fresh frozen plasma, *GI* gastro-intestinal, *RCT* randomized controlled trial

Many different 4-factor PCCs were used: Kanokad, Octaplex, Proplex T, Beriplex (in the USA used as Kcentra; hereafter referred to a Beriplex), PPSB-HT Nichiyaku, Kaskadil, Prothromplex and Cofact. Octaplex was the most often administered PCC. Four of the 4-factor PCC preparations contain a small amount of heparin (Beriplex, Octaplex, Kaskadil). Dosing of PCC was variable and included ‘fixed’ dose (n = 3), INR-based dosing (n = 2), weight-based dosing (n = 2) or a combination (n = 12). Six studies had good methodological quality, 9 were qualified as moderate, and 4 studies as poor.

### INR normalization

Of the 19 included studies, 16 reported on INR normalization (Table 2). The administered dose of PCC ranged from 5.3 to 80 IU/kg with a typical weight-based dose of 25–50 IU/kg.

**Table 2:** Indication and dosing of prothrombin complex concentrate, and effect on INR

References	Indication for PCC	PCC dose (range)	Baseline INR^a^	INR 0–15 min^a^	INR 15–30 min^a^	INR 1 (h)^a^	Comments
[34]	Melena: 3 (30%) Hematuria: 2 (20%) Other: 5 (50%)	30 IU/kg	>20 (8.9 to >20)	–	1.1 (1.0–1.3)	–	Median INR after 6–8 h: 1.1 24 h: 1.1 48 h: 1.6
[48]	ICH: 17 (100%)	500 or 1000 IU	2.7 (2.0 to >10)	1.1 (0.9–1.4)	–	–	–
[40]	GI: 5 (50%) Hematuria: 5 (50%)	14–44 IU/kg	Mean (SD): 7.1 (2.5)	Mean (SD): 1.8 (–)	Mean (SD): 1.8 (–)	Mean (SD) 1.9 (–)	–
[36]	Traumatic ICH: 111 (100%)	NA	–	–	–	–	Time to INR <1.5 PCC: 331 ± 279 min No treatment: 738 ± 692 min p value: *0.048*
[43]	GI: 8 (47%) Subcutaneous: 3 (18%) Other: 6 (35%)	35–50 IU/kg	4.8 (3.1–7.8)	–	1.1 (1.0–1.2)	–	–
[38]	ICH: 37 (100%)	500–1500 IU	PCC 2.29 (1.14–3.96) No treatment: 2.24 (1.11–4.23)	–	–	–	INR after PCC 2 h: 1.17 24 h: 1.14
[33]	ICH: 141 (100%)	1000 IU	2.6 (0.6–4.6)	–	–	1.4 (1.2–1.6)	INR <1.5 in 1 h: n = 101 6 h: n = 107
[31]	ICH: 82 (47%) Extracranial: 92 (53%)	21–25 IU/kg	ICH mean (SD): 4.1 (2.7) Extracranial mean (SD): 6.3 (3.9)	–	–	–	INR <1.5 in % patients ICH: 43 (52%) Extracranial: 35 (38%)
[31]	ICH: 300 (46%) GI: 167 (26%) Other: 179 (28%)	5.3–80 IU/kg	3.8	–	–	1.5 (0.9–2.1) in n = 163 (25%)	Target INR < 1.5 in n = 452 (70%)
[41]	ICH: 59 (51%) GI: 21 (18%) Other: 36 (31%)	18–29 IU/kg	3.5 (2.6–5.4)	–	1.4 (1.2–1.6)	–	–
[39]	ICH: 22 (100%)	15–25 IU/kg	Mean (SD): 4 (3)	–	–	–	INR after PCC Mean (SD): 1.3 (1.1) Time point unknown
[44]	GI: 127 (63%) Visible: 37 (18%) ICH: 24 (12%) Musculoskeletal: 14 (7%)	25, 35 or 50 IU/kg	PCC: 3.9 (1.8–20) FFP: 3.6 (1.9–38.9)	–	–	–	INR ≤ 1.3 in 0.5 h PCC: n = 61 (62%) FFP: n = 10 (9.6%) p value < *0.0001*
[46]	ICH: 22 (29%) Extracranial: 54 (71%)	1000 IU	2.8 (2.2–3.4)	–	–	–	Median INR 1.4 after 3 h
[42]	ICH: 135 (100%)	20–26 IU/kg	PCC: 3.0 (1.5–9.3) FFP: 2.9 (1.9–7.7)	–	–	–	75% of PCC patients received adequate dose to reduce INR < 1.5
[47]	Traumatic ICH: 29 (88%) Spontaneous ICH: 4 (12%)	25–50 IU/kg	PCC: 3.1 (–) FFP: 2.9 (–)	–	–	–	Time to INR < 1.6 PCC: 65 min FFP: 256 min
[37]	GI: 40 (100%)	25–50 IU/kg	PCC: 13.2 (4.5–21) FFP: 10 (3.0–21)	–	–	PCC 1.5 (1.1–2.3) FFP 4.5 (2.2–12.2)	INR after 6 h PCC: 1.5 (1.1–2.1) FFP: 2.4 (1.2–5.0)

^a^Median (range)

*ICH* intracranial hemorrhage, *IU/kg* international units per kilogram, *PCC* prothrombin complex concentrate, *FFP* fresh frozen plasma, *INR* international normalized ratio, *GI* gastro–intestinal, *NA* not applicable, *Min* minutes, *N* number of patients, *SD* standard deviation

Median baseline INR values ranged from 2.2 to higher than 20. In two studies the INR measurement was repeated within 15 min of PCC administration and the median INRs were 1.1 and 1.8 respectively. Thirty minutes after PCC administration, the INR ranged from 1.1 to 1.8 (n = 4) and after 1 h the range was 1.4–1.9 (n = 4). Time to INR <1.5 (<1.6 in one study) in the PCC groups ranged from 65 to 331 min. An hour after FFP administration the median INR was 4.5 (range 2.2–12.2) in one study. The INR normalized to <1.5 on average in 256 min in the FFP group (n = 1) and in 738 min in the no treatment group (n = 1).

Three studies compared INR normalization between PCC and FFP regimens. A prospective cohort study showed that the time to INR <1.6 was 65 min in patients treated with PCC versus 256 min in FFP treated patients [47]. Results of the RCT showed a significant reduction in time to INR normalization when PCC was used as compared to FFP (p < 0.0001) [44]. This was also observed in a prospective cohort study; after 1 h INR was normalized to 1.5 after PCC and to 4.5 after FFP administration [37]. In summary, PCC was able to reach INR correction more rapidly compared to FFP or no treatment.

### Mortality

Seventeen studies assessed mortality outcomes (Table 3). The time of follow-up ranged from 7 to 90 days in 10 studies, while the duration of follow-up for the remaining studies was not clearly reported.

**Table 3:** Interventions to treat VKA related bleeding, and functional and safety outcomes

Author	Intervention	Other interventions to stop bleeding	ICU admission (days)	Functional outcomes	Follow-up (days)	Mortality (n)	TE complications
[34]	PCC versus No control group	Vitamin K 10 (100%)	–	–	Unknown	0 (0%)	0 (0%)
[48]	PCC versus No control group	Vitamin K PCC: 13 (76%)	–	Modified Rankin scale 0–1: 6 (35%) 2–3: 5 (29%) 4–5: 3 (18%) 6: 3 (18%)	From admission to discharge	3 (18%)	–
[40]	PCC versus No control group	Vitamin K 3 (30%) Packed cells 3 (30%)	–	Response to treatment^a^ Good: 9 (90%) Moderate: 1 (10%)	Unknown	0 (0%)	–
[36]	PCC versus No treatment	Vitamin K PCC: 74% No treatment: 75%	PCC: 7.5 ± 6.3 No treatment: 5.8 ± 5.9 p value: *NS*	–	Unknown	PCC: 11 (24%) No treatment: 15 (23%) No OR calculated p value: *NS*	–
[38]	PCC versus No treatment	Vitamin K PCC: 15 (68%) No treatment: 14 (50%) FFP PCC: 1 (5%) No treatment: 5 (18%)	–	Modified Rankin scale ≥ 3 PCC: 10 (45%) No treatment: 20 (71%)	30	PCC: 1 (5%) No treatment: 7 (25%) No OR calculated	–
[33]	PCC versus No control group	Vitamin K 107 (85.6%) FFP 28 (22.4%)	–	Modified Rankin scale Median score 5	30	52 (37%)	7 (5%)
[31]	PCC versus No control group	Vitamin K 119 (68%) FFP 5 (3%) Packed cells 62 (36%)	–	–	7	0 (0%)	0 (0%)
[35]	PCC versus No treatment	Vitamin K PCC: 41 (100%) Vitamin K and/or FFP No treatment: 38 (27%)	–	–	90	PCC: 15 (37%) No treatment: 96 (69%) *HR 0.52 (0.29*–*0.93)* p value *0.029*	–
[31]	PCC versus No control group	Vitamin K 576 (89%)	–	–	15	135 (21%)	4 (0.6%)
[41]	PCC versus No control group	Unknown	–	–	7	7 (6%)	5 (4.3%)
[39]	PCC versus No control group	Unknown	–	–	Unknown	9 (43%)	–
[44]	PCC versus FFP	Packed cells PCC: 48 (49%) FFP: 47 (45%) Vitamin K PCC: 94 (96%) FFP: 102 (98%)	–	Hemostatic efficacy Excellent: PCC 44 versus FFP 45 Good: PCC 27 versus FFP 23 Poor: PCC 27 versus FFP 36	30	PCC: 6 (6.1%) FFP: 5 (4.8%) No OR calculated p value: *NS*	PCC: 8 (7.8%) FFP: 7 (6.4%) p value: *NS*
[46]	PCC versus No control group	–	–	Clinical efficacy outcomes Excellent: 61 Moderate: 5 None: 10	30	ICH: 7 (9%) EC: 8 (11%)	ICH: 6 (8%) EC: 14 (18%)
[42]	PCC versus FFP	Vitamin K All patients received vitamin K i.v. (100%)	–	–	30	PCC: 32 (32%) FFP: 19 (54%) *OR 0.40 (CI 0.18*–*0.87)* p value: *0.021*	–
[45]	PCC versus No treatment	Vitamin K 531 (65%) PCC + vitamin K 336 (41%)	–	–	7	110 (13%) of all patients ICH: 86 (33%) of patients with ICH	10 (1.2%)
[37]	PCC versus FFP	Vitamin K All patients received 10 mg vitamin K i.v. (100%)	PCC: n = 3 (15%) FFP: n = 4 (20%)	–	Unknown	PCC: 1 (5%) FFP: 1 (5%) *No OR calculated* p value: *NS*	–
[49]	PCC versus No control group	Vitamin K All patients (100%)	–	–	–	PCC: 10 (13%)	PCC: 0 (0%)

^a^Judged by investigators

*VKA* vitamin K antagonist, *PCC* prothrombin complex concentrate, *ICU* intensive care unit, *TE* thromboembolic, *FFP* fresh frozen plasma, *ICH* intracranial hemorrhage, *NS* not significant, *OR* odds ratio, *EC* extracranial, *I.V*. intravenous, *HR* hazard ratio

Overall 550 (19%) deaths were reported in 2828 patients. The mortality rate in the PCC group ranged from 0 to 43% [mean 17% (407/2436)], between 5 and 54% in FFP recipients [mean 16% (25/159)] and from 23 to 69% in the no treatment group [mean 51% (118/233)]. The mean mortality rates of patients treated with PCC and FFP were not statistically different (p = 0.73), whereas the mortality rate in the no treatment group was significantly higher than in the PCC group (p < 0.001).

### Thromboembolic complications

Nine studies reported on TE complications (Table 3). A total of 61 (2.7%) TE complications were observed in 2262 patients. The range of observed TE complication rate in the PCC group was 0–18% [mean 2.5% (54/2158)]. Only one study reported on TE complications in the FFP group with a mean of 6.4% (7/104). The mean TE complication rate did not differ between patients treated with PCC or FFP (p = 0.54). No data were available on TE complications in the no treatment group.

### Functional outcomes

Six studies evaluated functional outcomes (Table 3). Results of the included RCT showed similar functional outcomes between PCC and FFP on hemostatic efficacy, which is the rate of cessation of the bleeding assessed over a 24-h period after start of PCC [44]. Two studies without a control group assessed functional outcomes on the modified rankin scale (mRS) measuring the degree of disability or impairment of activities of daily life attributable to a ICH. In the first study a median mRS of 5 (severe disability) was observed in patients receiving PCC [33], and in the second 64% of PCC recipients had a mRS score of 5 or 6 [48]. Another study showed a mRS ≥ 3 (moderate to severe disability) in 10 (45%) of patients in the PCC group and in 20 (71%) of the patients in the no treatment group [38]. The other two studies did not used validated scores on functional outcomes, but showed good response to treatment [40] and excellent clinical outcomes [46].

### Exploratory meta-analysis

**Fig. 2:**
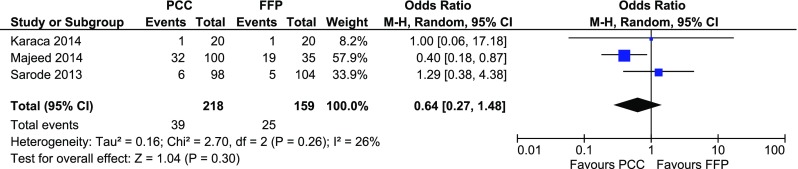
Forest plot comparison of mortality in patients treated with 4F-PCC versus FFP; *4F-PCC* 4-factor prothrombin complex concentrate; *FFP* fresh frozen plasma

The longest follow-up data from each of the included studies which incorporated mortality rates were used for the exploratory meta-analysis. Three studies [37, 42, 44] evaluated mortality in PCC versus FFP. The combined OR was 0.64 (95% CI 0.27–1.5; I^2^ = 26%; Fig. 2).

**Fig. 3:**
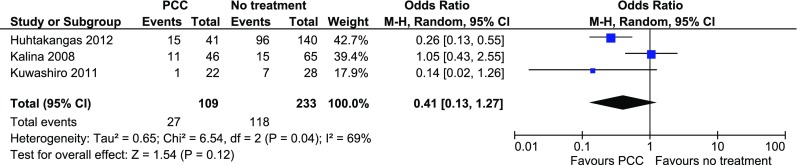
Forest plot comparison of mortality in patients treated with 4F-PCC versus no treatment. *4F-PCC* 4-factor prothrombin complex concentrate

Three other studies [35, 36, 38] compared PCC versus no treatment on overall mortality. Analysis of the pooled data showed an OR of 0.41 (95% CI 0.13–1.3; I^2^ = 69%; Fig. 3).

## Discussion

The results from the present analysis show that 4-factor PCC is a rapid and effective method to normalize INR in patients with VKA associated bleeding. PCC was shown to be more effective than FFP or no treatment in the correction of the INR to a level of 1.5 or lower. Our review also showed that mortality in patients treated with PCC was substantial, ranging from 0 to 43%, and was comparable to that in patients receiving FFP. Mortality rates were lower in the PCC group compared to no treatment. PCC therapy was associated with a low risk of TE complications, also in comparison to treatment with FFP, although based on small patient numbers from a single study. Finally, functional outcomes in PCC treated patients with VKA associated bleeding events were only assessed in a few studies and outcomes were highly variable.

To our knowledge this review is the first to describe a relatively homogeneous group of studies that all evaluated 4-factor PCC in patients with VKA associated bleeding complications (reversal for emergent interventions were not included). Moreover we included a substantial number of prospective studies and one RCT, with most of the studies of moderate or good methodological quality. Other strengths of the current project include the enhanced search strategy, the data collection and extraction by two independent researchers.

Similar findings regarding the efficacy of PCC in INR normalization have been observed in earlier reviews [6, 25, 26]. A recent Cochrane Review evaluated 4-factor PCC compared to administration of FFP in patients with VKA associated bleeding or indication for emergent procedures. The authors conclude that PCC is able to reverse VKA associated INR prolongation without further requiring FFP or other blood products [28].

The reported TE event rates from the current analysis are comparable to those from an earlier review in patients treated with 4-factor PCC [23] and another recent retrospective study evaluating the effects of 4-factor PCC [50]. In all analyses, including the present one, it is unclear in how many of the cases the PCC administration was directly related to TE events or is due to cessation of the anticoagulant treatment.

The observed mortality rate in the PCC group ranged from 0 to 43% with a mean of 17%. This rate is somewhat higher than reported in the literature in patients needing reversal with PCC for bleeding as well as surgery (11%) [23]. The high mortality rate in this review likely reflects the presence of severe intracranial bleeding in a large population of the included VKA users.

Some methodological aspects require comment. First, a limitation of our systematic review is that only one RCT could be included, while all other studies were cohort studies. As a result, causal relationships between 4-factior PCC and study outcomes cannot be made. Additionally, the results should be with interpreted with caution because in observational studies it is more likely that patients with a very poor predicted outcome at presentation receive less aggressive treatment, and therefore the patients that are selected to receive PCC have a better prognosis beforehand. Furthermore, also due to the observational nature of our data, the observed associations could possibly be explained by confounding or bias, and could therefore have overestimated or underestimated true treatment effects. To gain insight on the risk of bias in included studies, we assessed the methodological quality of these by means of validated tools. We found that in the vast majority of the studies the quality was moderate or high. Therefore we expect that the impact of bias on our study results will be limited. Secondly, in more than half of the studies there was no control group. Due to a limited number of patients in the control groups it is difficult to give a clear overview of the outcomes in efficacy between the PCC and the FFP/no treatment groups. Another aspect is the large variation in design, in- and exclusion criteria, treatment, definitions and representation of results. For example, differences in patient characteristics, PCC doses, INR standardization among laboratories, and baseline INR values between groups may have affected the response to PCC. Furthermore, the INR data provided by the included studies had different INR targets (INR <1.3–1.6) and reported on the INR outcomes in many different ways (Table 2). The large variability could have influenced the results. We tried to minimize this effect by executing a strict methodology by two researchers including a rigorous selection procedure. In addition, the results of the meta-analysis need to be interpreted with caution because they are based on an indirect comparison of studies that differed in sample size, inclusion criteria and methodological quality. Finally, vitamin K administration was not universally used in all studies, which should be kept in mind when interpreting the study outcomes.

Can these findings be translated into clinical practice? The patients included are real world patients experiencing major bleeding complications of VKA treatment. The results are therefore likely to be generalizable to other patients with VKA associated hemorrhages. However, a lot of other factors play a role, including the availability of 4-factor PCC, the resources of the hospital, the experience with VKA associated bleeding events etcetera. Current guidelines recommended the administration of PCC over FFP in patients with major bleeding, in addition to use of intravenous vitamin K [11] and our results subscribe this.

In conclusion, this study assessed clinical outcomes and laboratory measures of INR in patients with VKA-associated major bleeding events treated with 4-factor PCC. The most frequently observed bleeding was ICH. Our results indicate that 4-factor PCC is an effective and safe option for treatment of VKA associated bleeding complications. In addition, 4-factor PCC seemed to be more effective than FFP or no treatment in INR normalization without increasing the risk of TE complications and mortality.

## Electronic supplementary material

Below is the link to the electronic supplementary material.

Appendix. Full systematic search strategies. (PDF 21 KB)
