# Supplementary material for: Benefits and harms of 4-factor prothrombin complex concentrate for reversal of vitamin K antagonist associated bleeding: a systematic review and meta-analysis
Source: J Thromb Thrombolysis. 2017 May 24;44(1):118–29. doi: 10.1007/s11239-017-1506-0 (PMC5486892; doi:10.1007/s11239-017-1506-0)
Supplement: Supplementary file 1 — Appendix. Full systematic search strategies. (PDF 21 KB) [file 11239_2017_1506_MOESM1_ESM.pdf]

Appendix I. Full systematic search strategies.

PubMed.
Date of search: 20 August 2015.

(((("Vitamin K"[Mesh] OR vitamin K[tw] or VKA[tw] or Oral anticoagulant*[tw] or Anticoagulant*[tw] or Warfarin [tw] or Acenocoumarol[tw] or Sintrom[tw] or Sintrommitis[tw] or Phenprocoumon[tw] or Marcoumar[tw] or Coumarin[tw] or Coumarins[tw] or Coumarin derivate[tw] or Antivitamin K[tw])) AND ("Prothrombin"[Mesh] OR anticoagulant reversal[tw] or anticoagulation reversal[tw] or Prothrombin complex concentrate*[tw] or Complex concentrate*[tw] or Coagulation factor concentrate*[tw] or PCC[tw] or Cofact[tw] or Beriplex[tw] or Octaplex[tw] or kcentra[tw] or ppsb[tw]))) NOT (("Animals"[Mesh]) NOT "Humans"[Mesh])

Annotation: language limit applied (English, Dutch, French and German). MeSH indexed animal studies excluded.

Embase Classic + Embase
Date of search: 20 August 2015 via OvidSP**.**

1. anticoagulant agent/ or antivitamin k/ or exp coumarin anticoagulant/ or coumarin derivative/
2. (acenocoumarol or Sintrom or Sintrommitis or warfarin or vka or anticoagulant? or vitamin K or antivitamin? K or coumarin? or Phenprocoumon or Marcoumar).ab,kw,ti.
3. 1 or 2 [vitamin k antagonists]
4. prothrombin complex/
5. (pcc or prothrombin complex concentrate* or ppsb or Beriplex or Octaplex or kcentra or anticoagulant reversal or anticoagulation reversal or complex concentrate* or Coagulation factor concentrate* or cofact).ab,kw,ti.
6. 4 or 5 [PCC]
7. 3 and 6
8. (animal/ or animal experiment/ or animal model/ or nonhuman/ or rat/ or mouse/ or (rat or rats or mouse or mice).ti.) not human/
9. 7 not 8 [excl. animal studies]
10. limit 9 to (dutch or english or french or german)
11. remove duplicates from 10

Annotation: language limit applied (English, Dutch, French and German). Animal studies were exluded by a search string using Emtree index terms and title words.

Cochrane Central Register of Controlled Trials, Wiley Online Library
Date of search: 20 August 2015.

#1 vitamin K or VKA or Oral anticoagulant* or Anticoagulant* or Warfarin or Acenocoumarol or Sintrom or Sintrommitis or Phenprocoumon or Marcoumar or Coumarin or Coumarins or Coumarin derivate or Antivitamin K
#2 anticoagulant reversal or anticoagulation reversal or Prothrombin complex concentrate* or Complex concentrate* or Coagulation factor concentrate* or PCC or Cofact or Beriplex or Octaplex or kcentra or ppsb
#1 AND #2

Annotation: no additional limits applied, no animal studies excluded. Fields searched: title, abstract and keywords
